# Genetic ablation of *FASN* attenuates the invasive potential of prostate cancer driven by *Pten* loss

**DOI:** 10.1002/path.5587

**Published:** 2020-12-11

**Authors:** Débora C Bastos, Caroline F Ribeiro, Thomas Ahearn, Jéssica Nascimento, Hubert Pakula, John Clohessy, Lorelei Mucci, Thomas Roberts, Silvio M Zanata, Giorgia Zadra, Massimo Loda

**Affiliations:** ^1^ Department of Oncologic Pathology Dana‐Farber Cancer Institute Boston MA USA; ^2^ Department of Oral Biosciences University of Campinas Piracicaba Brazil; ^3^ Department of Pathology and Laboratory Medicine, Weill Cornell Medicine NewYork‐Presbyterian Hospital New York NY USA; ^4^ Department of Epidemiology Harvard T.H. Chan School of Public Health Boston MA USA; ^5^ Beth Israel Deaconess Medical Center Harvard Medical School Boston MA USA; ^6^ Department of Cancer Biology Dana‐Farber Cancer Institute Boston MA USA; ^7^ Departments of Basic Pathology and Cell Biology Universidade Federal do Paraná Curitiba Brazil; ^8^ New York Genome Center New York NY USA; ^9^ The Broad Institute Cambridge MA USA

**Keywords:** FASN, GEMM, invasion, prostate cancer, PTEN

## Abstract

Loss of the tumor suppressor gene *Pten* in murine prostate recapitulates human carcinogenesis and causes stromal proliferation surrounding murine prostate intraepithelial neoplasia (mPIN), which is reactive to microinvasion. In turn, invasion has been shown to be regulated in part by *de novo* fatty acid synthesis in prostate cancer. We therefore investigated the effects of genetic ablation of *Fasn* on invasive potential in prostate‐specific *Pten* knockout mice. Combined genetic ablation of *Fasn* and *Pten* reduced the weight and volume of all the prostate lobes when compared to single knockouts. The stromal reaction to microinvasion and the cell proliferation that typically occurs in *Pten* knockout were largely abolished by *Fasn* knockout. To verify that *Fasn* knockout indeed results in decreased invasive potential, we show that genetic ablation and pharmacologic inhibition of FASN in prostate cancer cells significantly inhibit cellular motility and invasion. Finally, combined loss of PTEN with FASN overexpression was associated with lethality as assessed in 660 prostate cancer patients with 14.2 years of median follow‐up. Taken together, these findings show that *de novo* lipogenesis contributes to the aggressive phenotype induced by *Pten* loss in murine prostate and targeting *Fasn* may reduce the invasive potential of prostate cancer driven by *Pten* loss. © 2020 The Authors. *The Journal of Pathology* published by John Wiley & Sons, Ltd. on behalf of The Pathological Society of Great Britain and Ireland.

## Introduction

Prostate cancer (PCa) is the second most common cancer in men worldwide and is the second leading cause of cancer death in American men (World Health Organization – Globocan, 2016). Deletions and mutations in the tumor suppressor gene phosphatase and tensin homologue (*PTEN*), which encodes the PTEN protein, are among the most frequent alterations found in prostate cancer, particularly in the metastatic setting [1, 2, 3]. Loss of *PTEN* expression is found in 17% of primary and 40.7% of metastatic prostate cancer and is associated with worse prognosis and shorter recurrence‐free survival [3, 4, 5, 6, 7, 8, 9], especially in ERG‐fusion negative samples [10, 11]. *PTEN* loss of activity due to mutations or deletions results in PIP3 accumulation and activation of the PI3K/AKT pathway [12, 13]. In fact, PI3K/AKT is the most frequently activated pathway in human malignant neoplasms, including prostate cancer [1, 10, 14].

Activation of PI3K/Akt signaling enhances invasive potential by modulating different targets in the cell, which facilitates prostate cancer progression. In human cells, *PTEN* overexpression decreases cell proliferation and migration due to reduced AKT phosphorylation [15]. Markers of invasion such as urokinase‐type plasminogen activator (uPA) and matrix metalloproteinase (MMP)‐9 are downregulated with *PTEN* expression or PI3K/AKT blockade [16]. In human and murine prostate cancer cells, increased expression of Cxcl12 and its receptor Cxcr4 follows PI3K/Akt activation, inducing cellular invasion and tumor growth [17]. Bone metastases are enhanced by angiogenesis induced by N‐cadherin through PI3K/AKT signaling [18], and by activation of the bone morphogenetic protein (BMP) signaling cascade through the PI3K/AKT–NF‐κB axis [19].

Fatty acid synthase (FASN) enzyme is an androgen‐regulated gene, responsible for *de novo* synthesis of long‐chain fatty acids. Lipid synthesis is essential for new cellular membrane synthesis required during cell division, for the post‐translational modification of signaling molecules, as well as for energy storage [20]. FASN facilitates oncogenesis when overexpressed in the mouse [21]. FASN is expressed at low to undetectable levels in normal tissues, except in breast during lactation, in liver, and in proliferative phase endometrium [22]. Also, non‐malignant cells typically acquire palmitate from the diet [23] and FASN inhibition minimally affects normal cells, including prostate [24]. On the other hand, elevated levels of FASN were found in prostates of transgenic mice of prostate adenocarcinoma (TRAMP) and its levels increased with age, tumor progression, and metastasis [25]. FASN is overexpressed in human prostate cancer, especially in the metastatic, castration‐resistant setting [26, 27, 28, 29, 30], and is associated with poor prognosis [29, 31]. The lipid biosynthesis process has been associated with invasion and metastasis. Inhibition of acetyl‐CoA carboxylase (ACC), another essential enzyme for the biosynthesis of fatty acids, decreases invadopodia formation and the ability of prostate cancer cells, among others, to invade [32]. In addition, *FASN* inhibition with siRNA in LNCaP cells reduces pseudopodia and invadopodia formation, cell adhesion, migration, and invasion [33]. Also, *FASN* inhibition with shRNA mediates actin cytoskeletal remodeling by decreasing the palmitoylation of Rho GTPases and the downstream activation of Cdc2, resulting in reduced prostate cancer cell migration [34]. Finally, *FASN* inhibition negatively regulates cytosolic phospholipase A2 (PLA2G4A) and estradiol 17‐beta‐dehydrogenase 12 (HSD17B12) in several prostate cancer cell lines. These enzymes, responsible for arachidonic acid and androgen production, respectively, when downregulated cause a decrease of downstream genes such as *RGS2*, *SPAG16*, *VWF*, and *RAP2B*, which are also involved in cellular motility, proliferation, and extracellular matrix interaction [33].

Somatic heterozygous deletion of *Pten* in mice results in prostatic intraepithelial neoplasia (PIN) by 10 months of age, with approximately 100% penetrance but not metastasis [35]. In contrast, prostate‐specific homozygous deletion of *Pten* results in PIN, which progresses to invasive carcinoma by 12–29 weeks. Lymph node metastases occur in 45% and lung metastases in 27% of the mice [35]. Overexpression of *FASN* and altered metabolism in prostate cancer cells are associated with inactivation of *PTEN* [28, 36, 37]; in contrast, *PTEN* expression is inversely correlated with *FASN* expression in prostate cancer [38], while inhibition of *PTEN* leads to the overexpression of *FASN in vitro*. Here, we characterize the role of *FASN* inactivation in modulating the invasive propensity of *PTEN* knockout (KO) in a genetically engineered modified mouse (GEMM) model.

## Materials and methods

### Mice

Animal studies were performed according to ARRIVE guidelines and approved by the Dana‐Farber Cancer Institute (DFCI) Institutional Animal Care and Use Committee (IACUC, Protocol number: 13‐014) in accordance with the NIH Guide for the Care and Use of Laboratory Animals; to the state and federal Animal Welfare Acts; and to institutional guidelines [Beth Israel Deaconess Medical Center (BIDMC) and DFCI]. All animals were maintained in an isolated environment in barrier cages. The generation of single and double KO mice for *Fasn* and *Pten* was conducted in three phases: colony establishment (phase I), generation of the experimental breeders (phase II), and generation of experimental cohorts (phase III). Phases I and II were conducted at the Preclinical Murine Pharmacogenetics Core Facility (Department of Medicine, BIDMC) and phase III was conducted at DFCI. Details are provided in the Supplementary materials and methods.

### Prostate tissue dissection and processing

At the endpoints (12 or 40 weeks), mice were weighed and euthanized by CO_2_ inhalation followed by cervical dislocation. Prostates were collected, weighed, and the anterior prostate (AP), dorso‐lateral prostate (DLP), and ventral prostate (VP) lobes were dissected as previously described [39], individually weighed and measured, and immediately fixed in 10% formaldehyde. Prostate tissues were paraffin‐embedded for histology and immunohistochemistry.

### Immunohistochemistry and Herovici's staining

Immunohistochemical staining of FASN, PTEN, p‐Akt, and Ki‐67 was performed on the Leica Bond Rx automated immunostainer (Leica Microsystems, Wetzlar, Germany). Details are provided in Supplementary materials and methods. Herovici's collagen staining was performed as recommended by the manufacturer (StatLab, McKinney, TX, USA) and described by Friend [40]. Briefly, following xylene and ethanol washes, slides were incubated with Herovici solution (to stain young collagen and reticulum blue, mature collagen red, and cytoplasm yellow), washed with 1% acetic acid, and incubated with Weigert's hematoxylin. Reactive stroma was calculated only in the prostate lobes of animals where *Fasn* KO occurred in more than 50% of the epithelial cells by measuring the area of stroma normalized by the total area of the lobe using ImageJ software (National Institutes of Health, Bethesda, MD, USA).

### Cell culture

The cells used for both migration and invasion assays (*Pten* ^+/−^) were isolated from prostate tissue of mice heterozygous for *Pten* loss. We followed an established protocol for digestion and single cell suspension [41], and cells were cultured in advanced DMEM/F12 medium (aDMEM/F12) supplemented with 10 mm HEPES, penicillin/streptomycin, 0.5 mm GlutaMAX, B27 Supplement (all from Thermo Fisher, Waltham, MA, USA), 1.25 mm *N*‐acetyl‐l‐cysteine (Sigma‐Aldrich, St Louis, MO, USA), 10 μm Y27632 (Tocris Bioscience, Bristol, UK), 250 nm A83‐01, 50 ng/ml EGF, 500 ng/ml R‐spondin 1, 100 ng/ml Noggin (all from PeproTech, Rocky Hill, NJ, USA), and 1 nm dihydrotestosterone (Sigma‐Aldrich). Cells were negative for mycoplasma, assessed using a colorimetric MycoAlert mycoplasma detection kit (Lonza, Walkersville, MD, USA).

### Wound healing assay and proliferation

*Pten* ^+/−^ cells (8 × 10^5^) were seeded in 24‐well plates in medium containing 500 nm IPI‐9119 (US Patents 8,546,432 and 9,346,769, developed by Infinity Pharmaceuticals, Cambridge, MA, USA) or vehicle (DMSO). After 20 h, confluent cell monolayers were scraped with a sterile 200‐μl pipette tip to create cell‐free areas. After three washes with PBS, medium containing the drug or vehicle was replaced and cells were incubated for 24 h. Images were obtained at 0 and 24 h and cell migration was analyzed with ImageJ. To confirm a specific FASN effect on migration, we also transfected *Pten* ^+/−^ cells for 24 h with 50 nm *Fasn* siRNA (#MSS274301 and #MSS236464; ThermoFisher Scientific, Waltham, MA,USA) and control siRNA (#12935300; ThermoFisher) before seeding cells as a confluent monolayer. Following wound formation, cells were imaged at 0 and 24 h (a total of 48 h incubation with siRNA). To exclude interference of proliferation, we concomitantly counted viable cells. Cells were plated as described and after 48 h were trypsinized and counted using the Vi Cell XR analyzer (Beckman Coulter, Fullerton, CA, USA) based on the trypan blue exclusion method.

### Boyden chamber assay

*Pten* ^+/−^ cells (8 × 10^5^) were either pretreated for 24 h with 500 nm IPI‐9119/DMSO or transfected for 24 h with 50 nm of *Fasn* siRNAs. Basement membrane‐coated inserts (CytoSelect™ CBA‐110; Cell Biolabs, San Diego, CA, USA) were incubated for 1 h at room temperature with aDMEM/F12 medium. Treated cells were suspended at 10^6^ cells/ml in 300 μl of supplemented aDMEM/F12 medium without serum and seeded onto the upper compartment of the inserts, with DMSO/IPI‐9119 or 50 nm siRNA. Medium containing 10% fetal bovine serum (500 μl) was added to the lower chamber and cells were allowed to invade for 2 days. Invasion was quantified by staining the fixed invasive cells (4% formaldehyde for 1 h at room temperature) with toluidine blue.

### Western blotting

To confirm FASN knockdown with siRNA, we perform a western blot [20] in the same conditions and at the same time points as the functional assays (proliferation, wound healing, and invasion). Forty‐eight and 72 h after siRNA incubation, *Pten* ^+/−^ cells were collected and lysed in RIPA buffer with the addition of phosphatase and protease inhibitor cocktail (Sigma‐Aldrich and Roche, Basel, Switzerland). Protein quantification was performed using the Bradford protein assay (Bio‐Rad, Hercules, CA, USA) and equal amounts of protein were resolved on precast Tris‐glycine polyacrylamide gels (Invitrogen). After transfer to nitrocellulose blotting membrane (Sigma), samples were incubated with primary antibodies to FASN (#3180; CST, Danvers, MA, USA) and vinculin (V9131; Sigma‐Aldrich).

### Study population

We included 660 men diagnosed with prostate cancer who were participants in the Physicians' Health Study (PHS) [42] or Health Professionals Follow‐Up Study (HPFS) [43]. The men were diagnosed between 1983 and 2004 and had archival prostate (prostatectomy and TURP) tumor materials for immunohistochemical evaluation. The PHS included two randomized trials investigating aspirin and vitamin supplements in the prevention of cardiovascular disease and cancer among a total of 29,071 male physicians, including 22,071 men aged 40–84 years starting in 1982 and an additional 7,000 men aged ≥50 years starting in 1999, with all receiving annual follow‐up questionnaires. The HPFS is an ongoing cohort of 51,529 male health professionals who have been followed with biennial questionnaires since 1986. Incident prostate cancers (ICD‐9: 185) were self‐reported and confirmed through medical record and pathology report review. Further details have been provided previously [44].

### Clinical and follow‐up data

We abstracted data on tumor stage, prostate‐specific antigen (PSA) level at diagnosis, and treatments from medical records and pathology reports. Standardized histopathologic review by study pathologists of H&E slides of each case provided uniform Gleason grading [45] as well as identification of areas of tumor for tissue microarray construction. Prostate cancer patients were followed up with written questionnaires to collect detailed information regarding treatments and clinical progression. For prostate cancer cases in the HPFS, treating physicians were contacted to collect clinical course information and to confirm development of metastases. For the PHS, self‐reported metastasis has been validated to be reported with high accuracy. Cause of death was determined by medical record and death certificate review. Follow‐up for mortality in the cohorts was greater than 98%.

### Statistical methods for human data

To evaluate if the association of tumor FASN with clinical outcomes is modified by PTEN status, we dichotomized protein expression of FASN based on the median expression as described by Nguyen *et al* [46] and cross‐classified dichotomized FASN and PTEN tumor status. For PTEN, we used the scoring and validation methods described previously [11]. Details are provided in Supplementary materials and methods.

## Results

### *Fasn* genetic ablation reduces prostate tumor progression mediated by *Pten* KO

**Figure 1:**
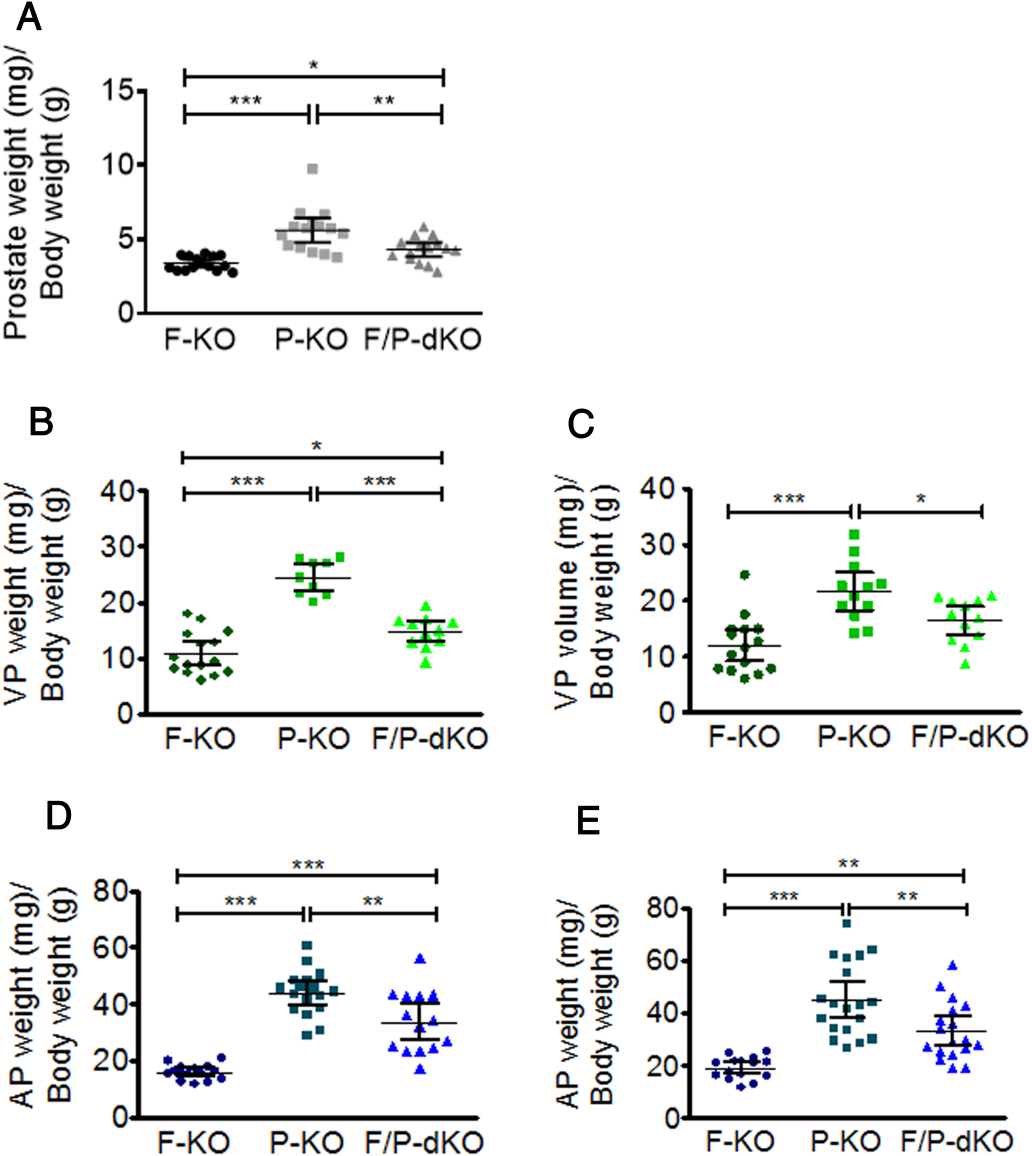
Prostate‐specific *Fasn* ablation (F‐KO) reduces the volume and/or weight of ventral and anterior prostate cancer induced by *Pten* loss (P‐KO) in 12‐week‐old mice. (A) Whole prostate weight. (B) Ventral prostate weight. (C) Ventral prostate volume. (D) Anterior prostate weight. (E) Anterior prostate volume. Prostate weights were normalized to body weight. *p < 0.01, **p < 0.001, ***p < 0.0001; ANOVA and Tukey's test. Error bars indicate mean ± SD (*n* = 15 mice).

Prostate‐specific loss of the tumor suppressor gene *Pten* results in spontaneous malignant lesions. To assess whether tumor progression induced by *Pten* KO is affected by *de novo* fatty acid production, heterozygous *Pten* and *Fasn* KO mice were crossed using a probasin Cre recombinase system and a breeding scheme was implemented as described in the Materials and methods section and supplementary material, Figures S1 and S2. The resulting *Pten* ^loxP/loxP^ *Fasn* ^wt/wt^Cre^pos^ (P‐KO), *Pten* ^wt/wt^ *Fasn* ^loxP/loxP^Cre^pos^ (F‐KO), and *Pten* ^loxP/loxP^ *Fasn* ^loxP/loxP^Cre^pos^ (F/P‐dKO) mice were analyzed at 12 and 40 weeks of age. After recording body weights, the prostate and its individual lobes (AP, DLP, and VP) were weighed, measured, photographed, and dissected (Figure 1A–E and supplementary material, Figure S3A–E). The specific deletion of *Fasn*, confirmed by IHC staining, decreased the weight and volume of the ventral lobes of 12‐week‐old (Figure 1B,C) and 40‐week‐old (supplementary material, Figure S3B,C) mice when compared with *Pten* single knockout mice. Similar results were observed for AP at 12 weeks (Figure 1D,E) but not at 40 weeks (supplementary material, Figure S3D,E). Only animals where *Fasn* KO occurred in more than 50% of the epithelial cells (supplementary material, Figure S4) were included in the analysis.

### Prostate‐specific *Fasn* KO reduces stromal reaction surrounding intraepithelial neoplasia and proliferation in *Pten* KO context

**Figure 2:**
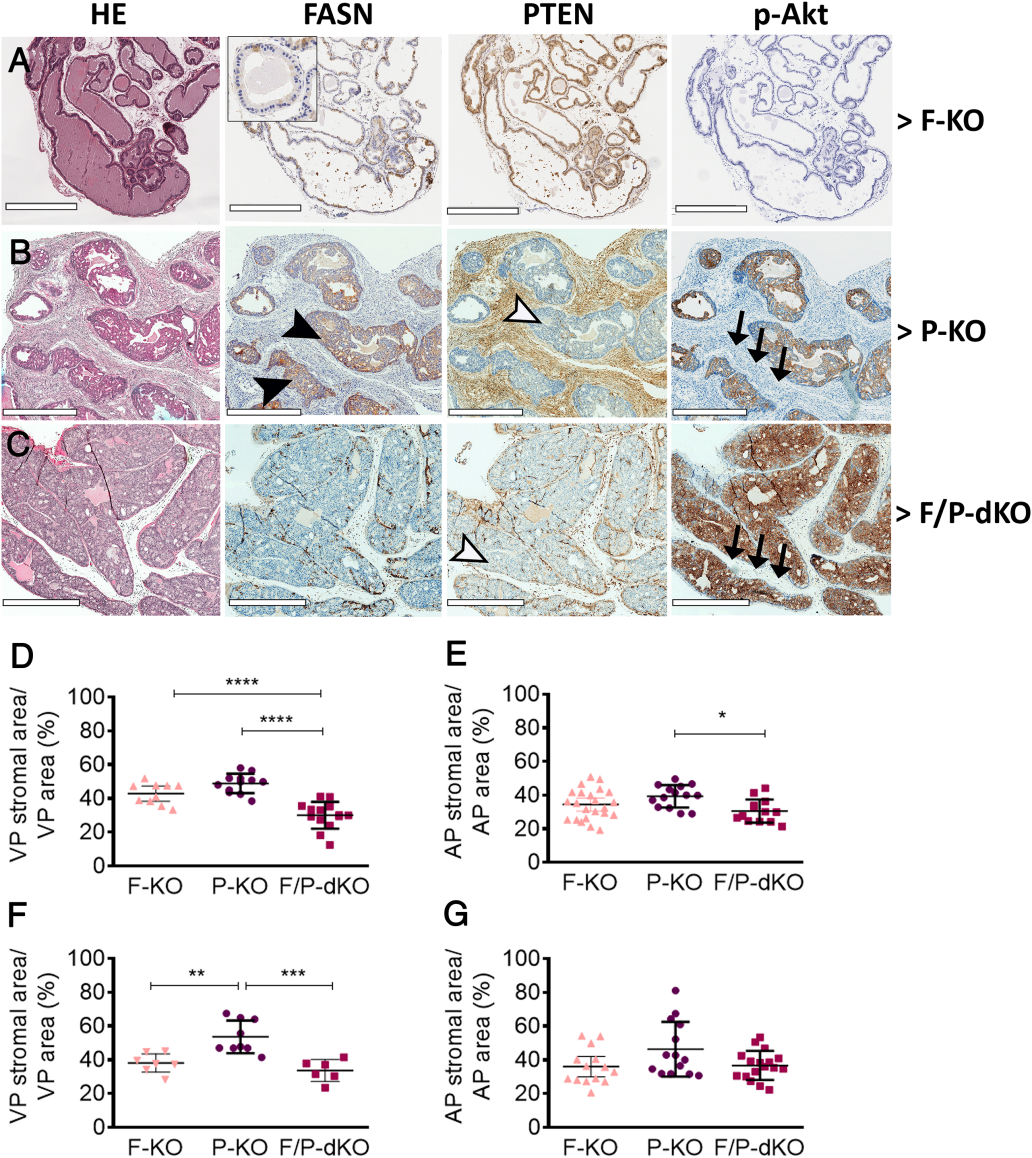
Conditional F‐KO affects prostate cancer progression through reduction of stromal and invasive areas in P‐KO mice. (A–C) H&E and immunohistochemistry images of the prostates of the indicated mouse strains. Black arrowheads indicate high levels of Fasn; white arrowheads indicate that Pten was deleted in the epithelial cells, but not in the stromal cells of P‐KO and F/P‐dKO mice; and black arrows indicate lack of p‐AKt in stromal cells. (D–E) Image analyses of stromal areas of 12‐week old mice and (F‐G) 40‐week old mice. *p < 0.05, **p < 0.01, ***p < 0.0001; unpaired *t*‐test. Scale bars = 500 μm (A) and 700 μm (B, C).

To investigate the reason for the decreased size and weight of prostate lobes in F/P‐dKO compared with P‐KO mice, histopathological analysis was performed in all prostate lobes and mouse genotypes that had been generated. As control, we confirmed that the prostates from the F‐KO group of 12‐week‐old (Figure 2A) and 40‐week‐old mice (supplementary material, Figure S5A) were histologically normal and, as expected, positive for PTEN but negative for p‐Akt staining. Also, we observed that whereas *Fasn* KO showed a chimeric pattern of inactivation (Figure 2A and supplementary material, Figure S5A), all epithelial cells in *Pten* KO mice exhibiting PIN were FASN‐positive (Figure 2A). Histopathological analysis of experimental groups revealed that the areas of PIN and stroma of anterior and ventral prostate lobes in P‐KO mice were 41.05% more extensive compared with F/P‐dKO mice (*p* < 0.0001).

**Figure 3:**
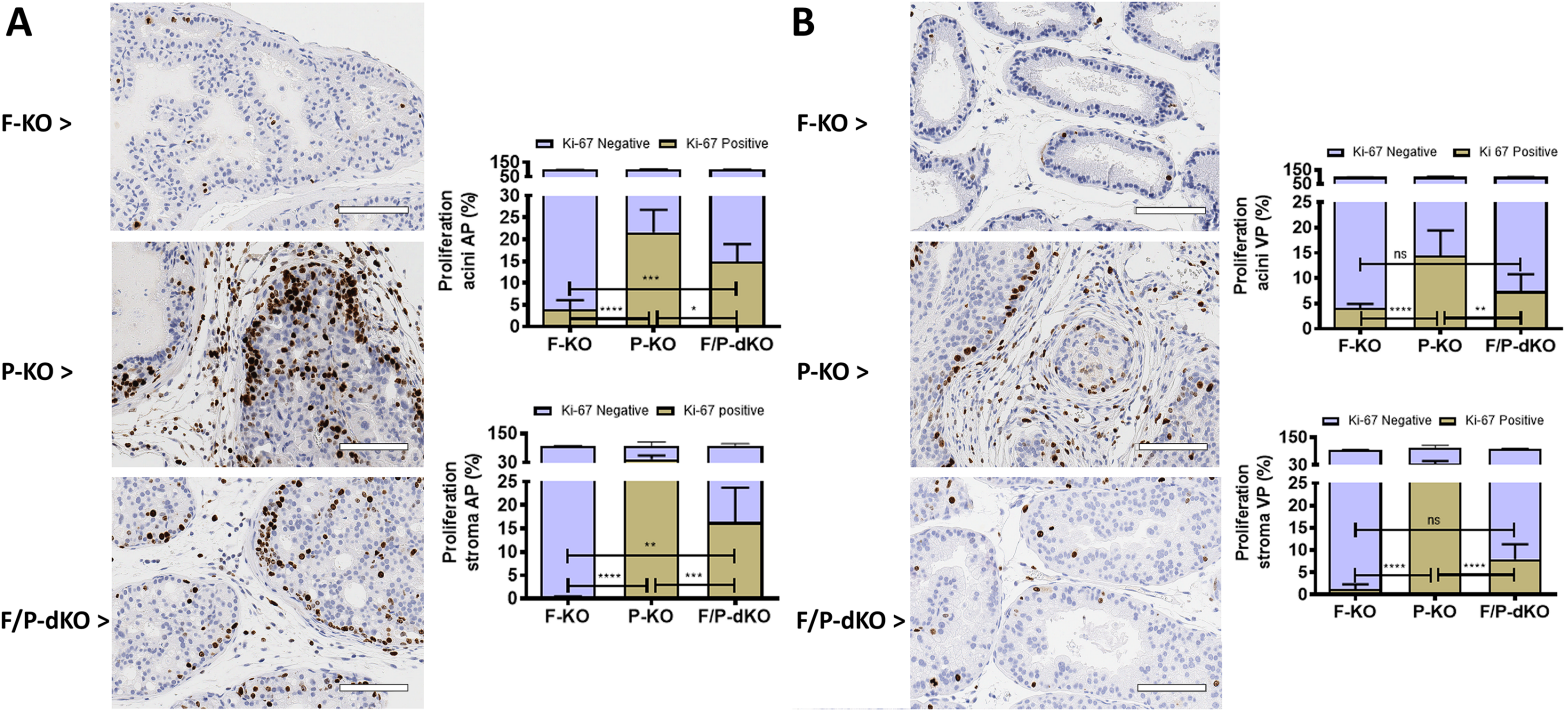
Ki‐67‐positive epithelial cells are reduced by the *Fasn* genetic ablation in P‐KO mice. (A, B) Ki‐67 staining and quantitation in 12‐week‐old mice. (A) Proliferation in the AP. (B) Proliferation in the VP. ns, not significant. *p < 0.05, **p < 0.005, ***p = 0.0001, ****p < 0.0001; ANOVA and Tukey's test. Scale bars = 100 μm.

We next studied the impact of FASN inactivation on the stromal reaction. We observed a dramatic difference in the quantity of reactive stroma when the ventral lobes of the P‐KO (Figure 2B) and F/P‐dKO groups (Figure 2C) were compared. The stromal areas in the VP and AP of 12‐week‐old (Figure 2D,E) and the VP of 40‐week‐old F/P‐dKO (Figure 2F,G and supplementary material, Figure S5C) mice were reduced in comparison to P‐KO mice (supplementary material, Figure S5B). Importantly, Ki‐67 positive staining of epithelial cells in the stroma was reduced by 27.7% and 26.1% in the AP (Figure 3A) and VP (Figure 3B), respectively, in the F/P‐dKO group when compared with P‐KO. On the other hand, proliferation in the acini was reduced by 6.7% and 7.2% in the AP and VP, respectively, in the F/P‐dKO group in comparison with P‐KO.

**Figure 4:**
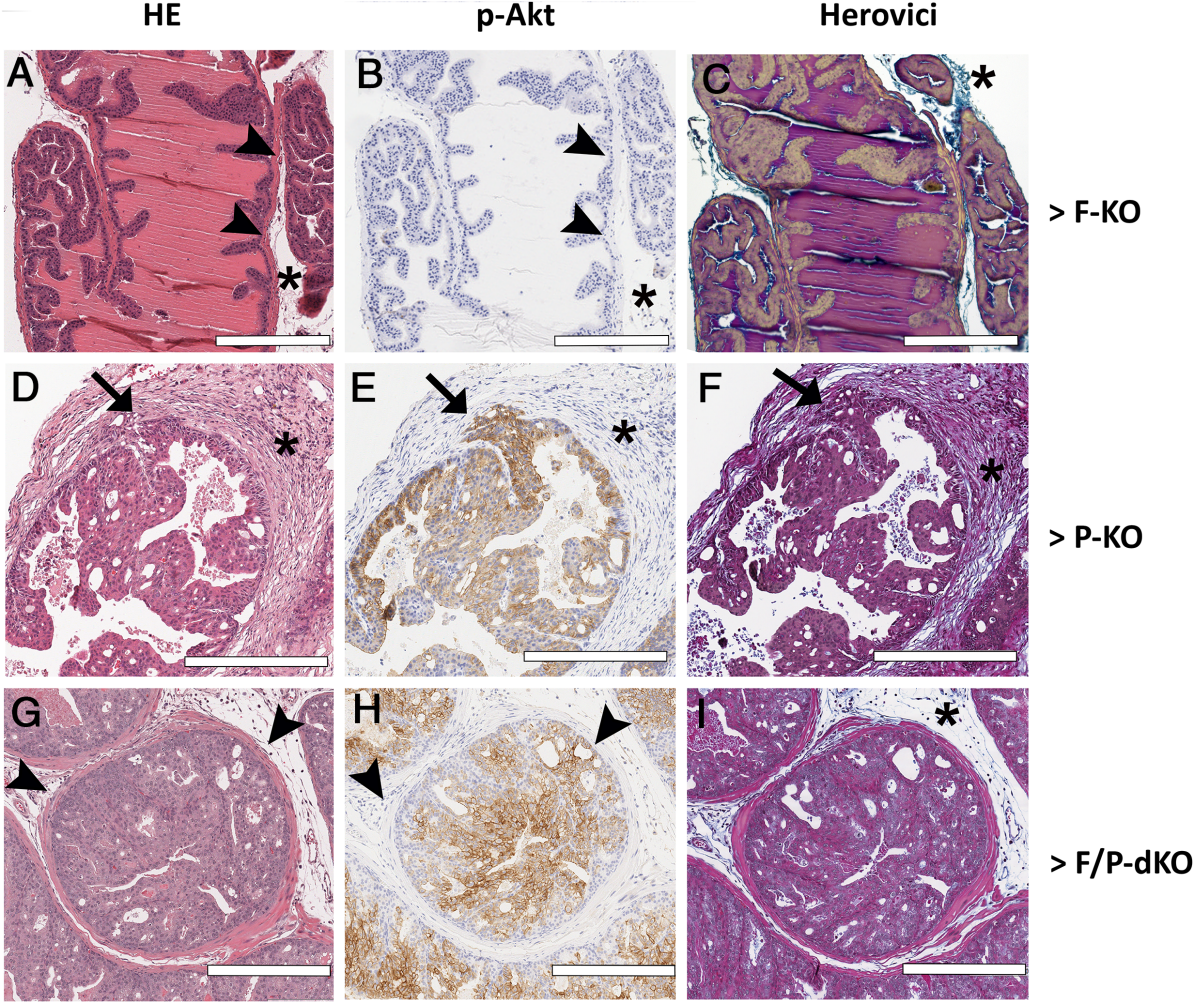
*Fasn* knockout reduced invasive areas in P‐KO mice. (A–I) H&E, p‐AKT immunohistochemistry and Herovici staining. Asterisk indicates stroma; arrowheads indicate the basal layer; and arrows indicate areas of invasion. Scale bars = 300 μm.

Several areas of microinvasion were identified in the P‐KO prostate (Figure 4D,E), while basal layer integrity was preserved in F‐KO (Figure 4A,B) and F/P‐dKO mice (Figure 4G,H). The Herovici stain (Figure 4C,F,I) demonstrated that the stroma of prostates in the F‐KO group (Figure 4C) was not reactive, devoid of *de novo* synthesized collagen (blue fibers), while the intense stromal reaction in the invasive areas of the P‐KO group (Figure 4C) is formed by new collagen fibers permeating old and mature collagen fibers (pink). On the other hand, we observed a sparse stroma in the F/P‐dKO group, formed by new collagen fibers between tumor islands that are surrounded by old and mature collagen fibers (Figure 4F), reflective of the loss or deceleration of the invasive phenotype.

### FASN depletion reduces PCa cell invasiveness

**Figure 5:**
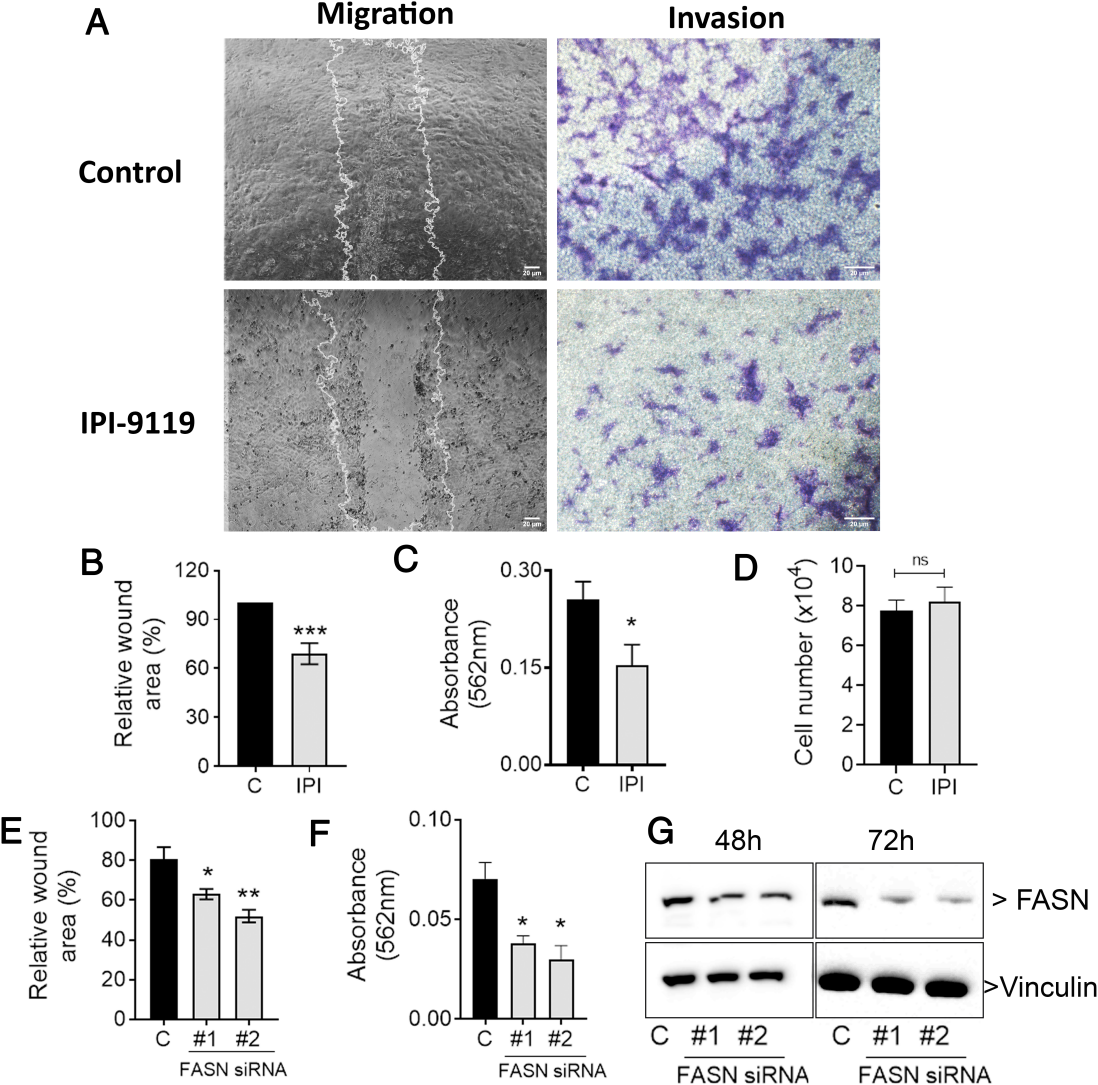
FASN depletion reduces the migration and invasion of murine epithelial PCa PTEN^pos/neg^ cell line. (A) Wound healing (right panel) and invasion through Boyden chambers coated with ECM (left panel) of PTEN^+/−^ prostate cancer cells treated with IPI‐119, quantified in B and C. (D) Cell viability for these data. (E, F) Wound healing and invasion of cells treated with *Fasn* siRNAs. (G) Representative immunoblotting for FASN in cells treated for 24 or 48 h with siRNAs. *p < 0.01, **p < 0.001, ***p < 0.0001; ANOVA and Tukey's test. Scale bars = 20 μm.

To confirm the negative effect of *Fasn* KO on the ability of prostate cancer cells to migrate and invade, we inhibited *de novo* fatty acid synthesis by genetic and pharmacologic means in *Pten* ^+/−^ prostate cancer cells. The selective FASN inhibitor IPI‐9119 [24] reduced both cell migration (Figure 5A, left panel, and 5B) and invasion (Figure 5A, right panel, and 5C) in *Pten* ^+/−^ prostate cancer cells, but not their viability (Figure 5D). Similar results were obtained with FASN knockdown by siRNA (Figure 5E,F), which was confirmed by western blot (Figure 5G).

### Combined *Pten* loss and FASN overexpression is associated with lethal prostate cancer

We analyzed two prospective cohorts of 660 incident prostate cancer cases diagnosed from 1983 to 2004 from the PHS [42] and HPFS [43]. Tumor FASN expression did not significantly differ by PTEN tumor status (*p* = 0.25). Clinical and demographic characteristics of the cohort by FASN/PTEN status are summarized in Table 1. Men with PTEN loss and high FASN tended to be diagnosed at an older age compared with other groups. Tumors with PTEN loss had higher Gleason grades and more advanced tumor stage irrespective of FASN expression. However, tumors with PTEN loss and high FASN tended to have the most advanced pathologic and clinical TNM stage. Mean body mass index did not differ across the FASN/PTEN groups.

**Table 1:** Association of cross‐classified FASN/PTEN with lethal prostate cancer among men diagnosed with prostate cancer between 1983 and 2004 in the Health Professionals Follow‐Up Study and the Physicians' Health Study.

	PTEN intact and FASN low (*n* = 267)	PTEN intact and FASN high (*n* = 262)	PTEN loss and FASN low (*n* = 63)	PTEN loss and FASN high (*n* = 68)
Mean age at diagnosis, years (SD)	65.9 (6.3)	65.2 (6.4)	65.0 (6.0)	66.8 (6.0)
Mean BMI at diagnosis, kg/m^2^ (SD)	25.8 (2.9)	25.7 (4.1)	25.5 (3.0)	25.1 (3.2)
Median PSA at diagnosis (Q1, Q3)*	7.4 (5.3, 11.0)	7.0 (5.0, 10.8)	6.9 (4.8, 10.5)	7.2 (5.0, 12.0)
Gleason grade, *n* (%)				
<6	45 (17)	54 (21)	5 (8)	5 (7)
3 + 4	107 (40)	111 (42)	14 (22)	16 (24)
4 + 3	67 (25)	56 (21)	22 (35)	25 (37)
8+	48 (18)	41 (16)	22 (35)	22 (33)
Pathologic TNM, *n* (%)				
T2 N0/Nx	169 (70)	186 (78)	27 (47)	38 (61)
T3 N0/Nx	69 (29)	48 (20)	29 (50)	17 (27)
T4 N1/M1	4 (2)	6 (3)	2 (3)	7 (11)
Clinical TNM, *n* (%)				
T1/T2	240 (93)	235 (92)	49 (80)	60 (92)
T3 N0/Nx	15 (6)	13 (5)	9 (15)	0 (0)
T4 N1/M1	3 (1)	7 (3)	3 (5)	5 (8)

* Cases with PSA at diagnosis: PTEN intact and FASN low = 221; PTEN intact and FASN high = 231; PTEN low and FASN low = 56; PTEN low and FASN high = 48.

**Figure 6:**
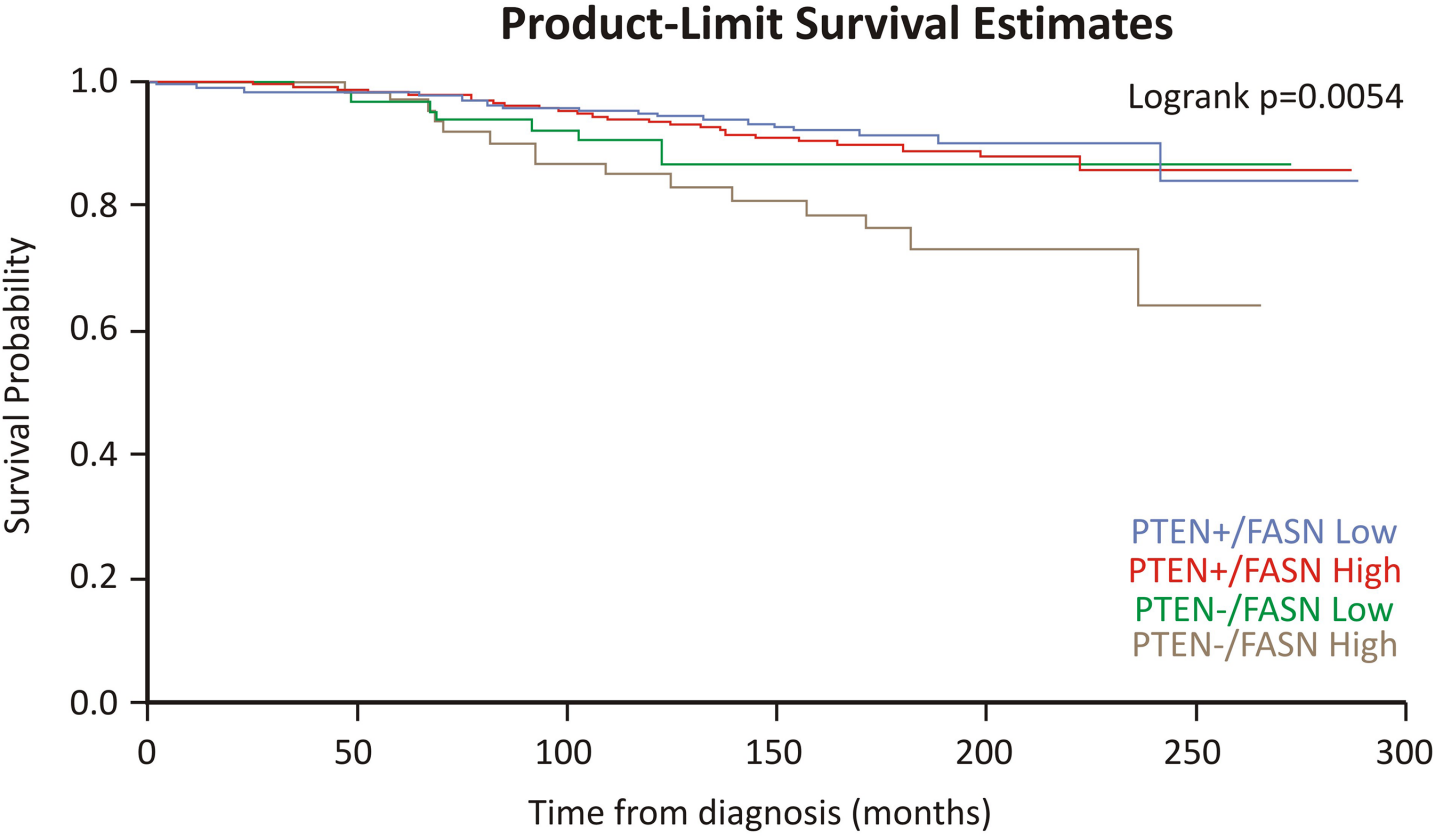
PTEN loss and FASN overexpression are associated with lethal prostate cancer. Kaplan–Meier survival plot for association of the interaction of FASN and PTEN status with lethal prostate cancer among men diagnosed with prostate cancer between 1983 and 2004 within the Health Professionals Follow‐Up Study and the Physicians' Health Study.

During a mean follow‐up of 14.2 years (range 0.11–25.8 years), 70 lethal events occurred. We evaluated and satisfied the proportional hazards assumption by testing the significance of the interaction between cross‐classified FASN/PTEN and follow‐up time in a model adjusting for age at diagnosis, Gleason grade, clinical TNM, and study cohort (Wald test *p* = 0.18). Table 2 and Figure 6 show the results of cross‐classified FASN/PTEN with lethal prostate cancer. In the multivariable models, tumors with PTEN loss and high FASN expression were associated with a higher risk for lethal progression (HR 2.0; 95% CI 1.1–3.9; *p* interaction = 0.03).

**Table 2:** Association of cross‐classified FASN/PTEN with lethal prostate cancer among men diagnosed with prostate cancer between 1983 and 2004 in the Health Professionals Follow‐Up Study and the Physicians' Health Study.

PTEN/FASN expression	*N*	Lethal	HR (95% CI)*	*p*_int_ ^†^
Any PTEN intact^‡^/FASN low	267	23	Reference	0.03
Any PTEN intact^‡^/FASN high	262	24	0.9 (0.5–1.6)	
Complete PTEN loss^**§**^/FASN low	63	7	0.6 (0.3–1.5)	
Complete PTEN loss^**§**^/FASN high	68	16	2.0 (1.1–3.9)	

* Adjusted for age at diagnosis, cTNM, and Gleason grade.

^†^ *P* value of PTEN and FASN multiplicative interaction based on the Wald test.

^‡^ At least one TMA core with PTEN intact.

^§^ All TMA cores with PTEN loss.

## Discussion

The tumor suppressor gene *PTEN* has been extensively associated with prostate cancer development and progression [2, 3, 4, 6, 7, 8, 9]. Metabolic changes also occur during prostate tumorigenesis, including alterations in *de novo* lipogenesis [37, 47, 48]. Increased levels of FASN are observed in the early phase of prostatic carcinogenesis and its overexpression is associated with poor prognosis, metastasis, and chemoresistance [21, 26, 28, 29, 49, 50].

Several studies have reported that pharmacological or genetic FASN inhibition results in cell cycle arrest, apoptosis, and reduction of prostate cancer progression in xenograft models [24, 47, 51, 52]. Previous studies in prostate cancer were mostly conducted with FASN inhibitors such as C75, orlistat, and triclosan [53, 54, 55], whose off‐target effects as well as instability and poor solubility/oral availability have limited their use in the clinical setting [56]. However, we recently demonstrated that IPI‐9119, a specific and selective inhibitor of FASN, impairs the growth of mCRPC xenografts and human organoids while inducing substantial metabolic reprogramming [24]. In addition, FASN inhibition with TVB‐3166 reduced both tubulin palmitoylation and expression, and the combination TVB‐3166/paclitaxel decreased prostate (22Rv1) xenograft growth [51]. TVB‐2640 (ASC‐40, Alcletis and 3V‐Bioscience), which shares molecular similarities with TVB‐3166, is being used in a phase I clinical trial for patients with solid tumors (NCT02223247) as well as in phase II trials for colon cancer (NCT02980029), KRAS non‐small cell lung carcinomas (NCT03808558), astrocytomas (NCT03032484), and ErbB2‐positive breast cancer (NCT03179904).

Here, we sought to gain insights into the mechanism responsible for halting progression of castration‐resistant prostate tumors with a deficient *Pten* background. First, we observed that prostate‐specific *Fasn* deletion is associated with a significant reduction of the stromal area surrounding expanding PIN lesions induced by *Pten* KO. Stromal reaction is likely associated with microinvasion and is typical in P‐KO tumors as they advance. Prostate lobes were in fact hardened and enlarged in P‐KO mice compared with F/P‐KO mice. Inhibition of lipid metabolism has been associated with decreased cellular migration and invasion in several cancer cell lines [57, 58, 59, 60, 61, 62, 63]. In addition, the stroma may play a significant role in tumor progression [64] and contributes to gland enlargement and tumor weight. Thus, lobe weighing is useful to indirectly assess tumor progression. Acini in P‐KO mice are spaced far apart and occupy proportionally less space compared with F/P‐KO. Also, proliferation of epithelial cells dispersed in the stroma was reduced in F/P‐dKO compared with P‐KO. Although the reduction of epithelial cell proliferation in acini was also significant in the F/P‐dKO group, this reduction was less pronounced compared with the Ki‐67 staining reduction in the stroma.

These results suggest that prostate‐specific FASN inactivation affects cancer progression in the early phases of carcinogenesis (12‐week‐old mice) and that these effects are maintained during tumor progression (40‐week‐old mice), especially in the VP. In general, effects on weight, volume, and stromal reaction were more pronounced in the VP than in the AP, especially at 40 weeks. We attribute this to the following reasons: the commonly observed accumulation of liquids in 40‐week‐old mouse AP, which affects the weight, size, and distance between the acini in the AP, but does not occur in the VP; that the stroma of normal VP is thinner and more delicate than the prominent connective tissue in the stroma of normal AP [65] makes even subtle changes more easily identified in the VP. Consistent with these findings, we have previously shown that knockout of both PI3K mediators (p110 alpha and beta) is required to reverse PIN in the VP, while the loss of p110 beta alone is needed to reverse it in the AP [66]. Thus, the VP is biologically the lobe that requires full PI3K signaling in tumorigenesis and is therefore the more appropriate lobe to assess the differences that we observed. Additionally, it is important to highlight that prostates of F‐KO mice were histologically normal, positive for PTEN, and negative for p‐Akt staining. Our group has been working with *Fasn* KO mice for many years [67] and we have found no histological differences between prostate, or other organs, in WT and *Fasn* KO mice of the same genetic background at all ages (unpublished data). In addition, it is widely reported that pharmacologic inhibition of FASN minimally affects normal prostate cells [24]. Also, we observed that whereas F‐KO showed a chimeric pattern of inactivation in epithelial cells, PIN lesions arising in P‐KO are all FASN‐positive.

Inhibition of acetyl‐CoA carboxylase (ACC) decreases invadopodia formation and the ability of prostate cancer cells to invade [32]. In addition, *FASN* inactivation with siRNA in LNCaP cells reduces pseudopodia and invadopodia formation, cell adhesion, migration and invasion, and *FASN* inhibition negatively regulates PLA2G4A and HSD17B12 in several prostate cancer cell lines [33]. Here, we demonstrated that FASN inhibits tumor progression by reducing the migration and invasion of *Pten* heterozygous prostate cancer cells. Further, *in vitro Fasn* inactivation by shRNA affects the adhesion, migration, and invasion of prostate cancer cell lines via actin cytoskeletal remodeling, achieved by decreasing the palmitoylation of the GTPase RhoU and consequent inactivation of Cdc42 [34].

FASN overexpression was previously associated with *PTEN* inactivation [28]. Bandyopadhyay *et al* [38] demonstrated that inhibition of the tumor suppressor gene *Pten* was inversely correlated with FASN expression in prostate cancer in the clinical setting. In that study, 43 patients with follow‐up of 5 years were examined. Inhibition of *PTEN* led to the overexpression of *FASN in vitro* [27]. Here, we validated and extended these data in a cohort of 660 prostate cancer patients with median follow‐up of 14.2 years, sufficient to adequately assess outcome [68]. Furthermore, we correlated PTEN and FASN with clinical characteristics (body mass index, PSA levels, Gleason grade, and pathological and clinical TNM). Interestingly, FASN did not differ by PTEN status, but tumors with PTEN loss and high FASN expression had the most advanced pathologic and clinical TNM stage. Importantly, in the multivariate models, tumors with PTEN loss and high FASN levels were associated with a higher risk for lethal progression. Considering that the new FASN inhibitor TVB‐2640 is the first‐in‐class in clinical trials, the data provide a rationale for utilizing FASN inhibitors in the treatment of prostate cancer, especially those with *Pten* loss.

In conclusion, we demonstrate that *Fasn* genetic ablation reduces invasive potential in the well‐established prostate‐specific *Pten* knockout mouse model, which arguably reflects one of the most frequent genetic alterations in human prostate cancer [1]. The reduction in stromal reaction induced by invading *Pten*‐null cells suggests that *de novo* fatty acid synthesis is required for the invasive and migratory potential of *Pten*‐loss prostate cancer cells. Thus, targeting lipid metabolism represents an attractive therapeutic strategy in advanced prostate cancer, especially in tumors harboring *Pten* loss.

## Author contributions statement

DCB, GZ and ML designed the research study. DCB, CFR, TA, JN, HP and JC conceived and carried out experiments. LM and TR provided human data analysis. DCB, CFR, GZ, SMZ and ML analyzed and interpreted the data. DCB, CFR, GZ and ML wrote and/or reviewed the manuscript. All the authors gave final approval of the submitted and published versions.

## Supporting information


**Supplementary materials and methods**
**Figure S1.** Breeding scheme for P‐KO, F‐KO, and F/P‐dKO mouse generation
**Figure S2.** *Pten* and *Fasn* prostate‐specific deletion
**Figure S3.** Prostate weights, volumes and stromal areas at 40 weeks
**Figure S4**. Discriminating Fasn‐positive and ‐negative regions
**Figure S5.** Histological and immunohistochemical aspects of 40‐week‐old prostates
**Table S1.** Summary of the primers and PCR conditions used
