# Supplementary material for: Genetic ablation of FASN attenuates the invasive potential of prostate cancer driven by Pten loss
Source: J Pathol. 2020 Dec 11;253(3):292–303. doi: 10.1002/path.5587 (PMC7898611; doi:10.1002/path.5587)
Supplement: Supplementary file 1 — Supplementary materials and methods Figure S1. Breeding scheme for P‐KO, F‐KO, and F/P‐dKO mouse generation Figure S2. Pten and Fasn prostate‐specific deletion Figure S3. Prostate weights, volumes and stromal areas at 40 weeks Figure S4. Discriminating Fasn‐positive and ‐negative regions Figure S5. Histological and immunohistochemical aspects of 40‐week‐old prostates Table S1. Summary of the primers and PCR conditions used [file PATH-253-292-s001.docx]

**Genetic ablation of *FASN* attenuates the invasive potential of prostate cancer driven by *Pten* loss**

DC Bastos, CF Ribeiro *et al. J Pathol* DOI: 10.1002/path.5587

**Supplementary materials and methods**

**Supplementary Figures S1–S5**

**Supplementary Table S1**

Reference numbers refer to the main text list

**Supplementary materials and methods**

*Generation of mice*

The breeding followed the monogamous mating scheme and the male was separated from the female after the confirmation of pregnancy. After weaning, tail tips were used for DNA extraction and genotyping. At the time of tail biopsy, the animals were identified by ear punch and monitored at least twice weekly (body weight, palpation, and body condition scoring) throughout their lifespan. The generation of the single and double knockout mice for FASN and PTEN was conducted in three phases: colony establishment (phase I), generation of the experimental breeders (phase II), and generation of experimental cohorts (phase III). Phase I consisted in the generation of heterozygous mice for *Fasn* and *Pten* (*Fasn*^loxP/wt^*Pten*^loxP/wt^Cre^pos^ males and *Fasn*^loxP/wt^*Pten*^loxP/wt^Cre^neg^ females) by crossing *Pten*^loxP/loxP^*Fasn*^wt/wt^Cre^pos^ mice [69] with *Fasn*^loxP/loxP^*Pten*^wt/wt^Cre^neg^ mice, kindly provided by Dr Semenkovich’s lab (Department of Medicine, Washington University School of Medicine). For phase II, heterozygous male and female mice generated in phase I (supplementary material, Figure S1A) were crossed to obtain the breeders for each cohort: the P-KO cohort (males *Pten*^loxP/loxP^*Fasn*^wt/wt^Cre^pos^ and females *Pten*^loxP/loxP^*Fasn*^wt/wt^Cre^neg^), F-KO cohort (males *Fasn*^loxP/loxP^*Pten*^wt/wt^Cre^pos^ and females *Pten*^loxP/loxP^*Fasn*^wt/wt^Cre^neg^), and F/P-dKO cohort (males *Pten*^loxP/loxP^*Fasn*^loxP/loxP^Cre^pos^ and females *Pten*^loxP/loxP^*Fasn*^loxP/loxP^Cre^neg^).

In phase III, males and females for each cohort obtained in phase II were crossed to increase the number of males for each group: P-KO cohort (supplementary material, Figure S1B), F-KO cohort (supplementary material, Figure S1C), and F/P-dKO cohort (supplementary material, Figure S1D). Near the endpoints, the animals were monitored daily. In our model, the phosphatase domain of *Pten* was inactivated by lox-flanking exon 5 of *Pten* in C57Bl/6 female mice (supplementary material, Figure S2A). Also, Cre recombinase expression is under the control of a modified rat prostate-specific probasin promoter, ARR2Probasin-Cre transgenic line (supplementary material, Figure S2B), as previously reported by work performed at Dr Chen’s lab – Department of Cancer Biology, Wake Forest University School of Medicine [70–72]. Prostate-specific *Pten* inactivation occurs due to the formation of a truncated Pten protein (supplementary material, Figure S2C). Indeed, *Fasn* prostate-specific deletion inactivation occurs due to partial deletion of the β-ketoacyl-synthase domain by lox-flanking exons 4–8 (808–1556 bp) of *Fasn* in C57Bl/6 female mice (supplementary material, Figure S2D) and crossing them with heterozygous ARR2PbCre C57Bl/6 males (supplementary material, Figure S2E) as described in the establishment of colonies (phase I). Prostate-specific deletion of exons 4–8 results in truncated Fasn by the insertion of a stop codon at 1592 bp (supplementary material, Figure S2F). The truncated FASN has 104 amino acids, whereas the murine FASN has about 2500 amino acids.

*Genotyping*

DNA from tails was extracted using a Gentra Puregene Mouse Tail Kit (Qiagen, Minneapolis, MN, USA) following the manufacturer’s instructions. Mice were genotyped by PCR using FASN^loxP^, PTEN^loxP^, and PbCre mouse-specific primers and conditions described in supplementary material, Table S1.

*Immunohistochemistry*

The sections were deparaffinized and antigen retrieval was carried out with Bond Epitope Retrieval Solution 1 or 2 (for Ki-67), pH 6.0 (Leica Microsystems, Bannockburn, IL, USA), for 20 min. The slides were incubated with rabbit anti-FASN (Cat No A301-324A; Bethyl Laboratories, Montgomery, TX, USA) at a dilution of 1:1000, rabbit anti-PTEN (Cat No 9188; Cell Signaling, Danvers, MA, USA) at a dilution of 1:200, rabbit anti-pAkt (Cat No 4060; Cell Signaling) at a dilution of 1:50, and rabbit anti-Ki67 (Cat No ab16667; Abcam, Cambridge, MA, USA) at a dilution of 1:100 for 60 min. The sections were treated according to the streptavidin–biotin–peroxidase complex method (Bond Polymer Refine Detection, Leica Microsystems) with diaminobenzidine (DAB) as chromogen and counterstained with hematoxylin. For human tissues, immunohistochemistry was performed using tumor tissue available from a biorepository of archival radical prostatectomy (RP, 95%) and transurethral resection of the prostate (TURP, 5%) tumor specimens. Hematoxylin and eosin slides were reviewed by our study pathologists to confirm prostate cancer and to identify tumor areas for tissue microarray (TMA) construction. Fourteen TMAs were constructed by sampling at least three 0.6 mm cores of tumor per case from the dominant nodule or the nodule with the highest Gleason pattern. Five-micrometer sections of each TMA were deparaffinized in xylene, followed by graded alcohol rehydration. The methods describing the IHC staining and scoring protocols for FASN and PTEN have been previously described [4,21]. Briefly, FASN IHC was performed using a rabbit anti-FASN (1:200 dilution; Assay Designs, Ann Harbor, MI, USA). Image analysis was conducted using an Ariol SL-50 instrument (Applied Imaging, Grand Rapids, MI, USA) to score FASN expression. Normal prostatic glands were excluded from analysis by manual circling. The FASN mean region score [positive area/(positive + negative area)] and the mean positive intensity across tumor cores were calculated. FASN expression was summarized by multiplying the mean region score by the mean intensity. For PTEN staining, antigen retrieval was performed by steaming in EDTA buffer (pH 8.0) for 45 min. Endogenous peroxidase activity was quenched by incubation with peroxidase block for 5 min at room temperature. Slides were incubated for 45 min at room temperature with rabbit anti-human PTEN (Cat No 9188; Cell Signaling; 1:50 dilution). A horseradish peroxidase-labeled polymer (PowerVision, Leica Microsystems) was applied for 30 min at room temperature and signal detection was performed using DAB as chromogen. Slides were counterstained with hematoxylin, dehydrated, and mounted. A tissue core was considered to have PTEN protein loss if the intensity of cytoplasmic and nuclear staining was entirely lost (0+ intensity) or markedly decreased (1+ intensity) across >10% of tumor cells compared with surrounding benign glands and/or stroma, which provide internal positive controls for PTEN protein expression.

*Statistical methods for human data*

Briefly, FASN scoring was determined by image analysis using two output variables combined to obtain a single FASN protein expression score: percentage of positive cells (positive area/positive negative area) and the numerical measure of FASN staining intensity. PTEN double-blinded scoring was conducted by the study pathologists. PTEN inactivation was determined by the complete loss of cytoplasmic and nuclear staining (0+ intensity) or markedly decreased (1+ intensity) in more than 10% of tumor cells compared with surrounding benign glands and/or stroma compared with positive controls for PTEN. We evaluated the association of demographic, clinical characteristics and prognostic outcomes with cross-classified FASN/PTEN. We used Cox proportional hazards models to estimate hazard ratios (HRs) and 95% confidence intervals (CIs) to test if the combination of high FASN and PTEN loss was associated with an increased risk of lethal prostate cancer (defined as prostate cancer death or metastases to bone or other organs), relative to men with low tumor FASN expression and intact PTEN. Person-time was calculated from the date of cancer diagnosis to the earliest of the following time points: development of lethal prostate cancer, censored at time of death from other causes, or end of follow-up (December 2014 for the PHS and December–March 2015 for the HPFS). Models were adjusted for age at diagnosis (years, continuous), study (HPHS or PHS), Gleason grade (≤6, 3 + 4, 4 + 3, 8–10; ordinal), and clinical stage (T1/T2 N0/Nx M0/Mx, T3 N0/Nx M0/Mx, and T4 N1 M1; ordinal). As a sensitivity analysis, we restricted to men with radical prostatectomy tissue (615 cases) and adjusted for pathologic stage in place of clinical stage. The Kaplan–Meier method was used to estimate survival probabilities and the log-rank test to compare groups. For all models, the proportional hazards assumption was evaluated and satisfied by testing the significance of the interaction between cross-classified FASN/PTEN and follow-up time.

**Supplementary Figures S1–S5**


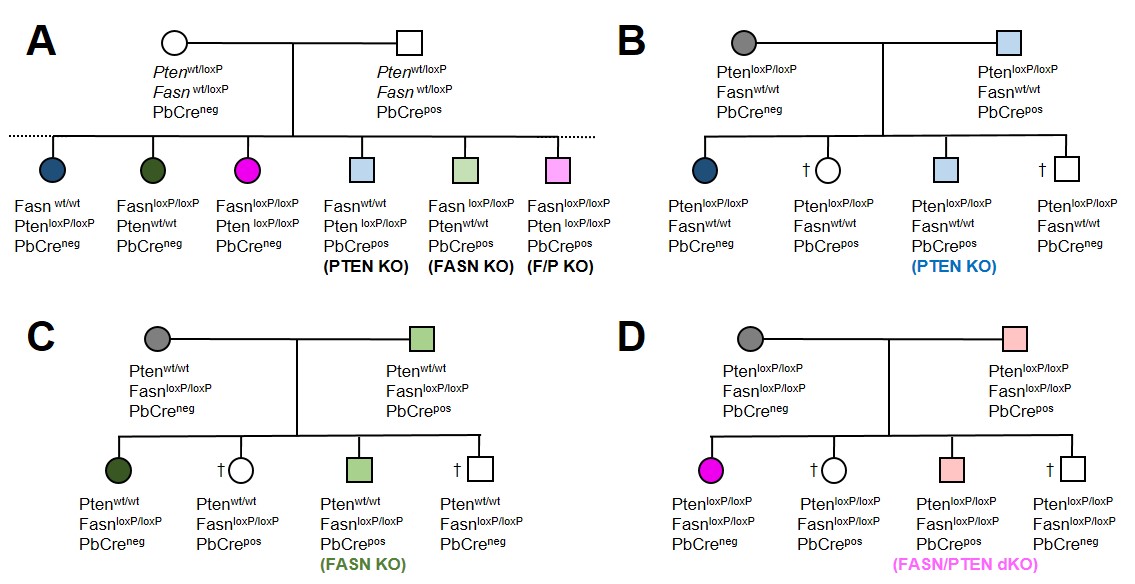


**Figure S1.** Breeding scheme for P-KO, F-KO, and F/P-dKO mouse generation. (A) The breeders (blue = P-KO; green = F-KO; pink = F/P-dKO) for each group were generated with a frequency of 1/64 by crossing heterozygous *Pten*/*Fasn* PbCre^neg^ females with heterozygous *Pten*/*Fasn* PbCre^pos^ males. (B) Prostate-specific P-KO males were obtained with a frequency of 1/4 by crossing homozygous *Pten*^loxP/loxP^/*Fasn*^wt/wt^ PbCre^neg^ females with *Pten*^loxP/loxP^/*Fasn*^wt/wt^ PbCre^pos^ males. (C) Prostate-specific F-KO males were obtained with a frequency of 1/4 by crossing *Pten*^wt/wt^/*Fasn*^loxP/loxP^ PbCre^neg^ females with *Pten*^wt/wt^/*Fasn*^loxP/loxP^ PbCre^pos^ males. (D) Prostate-specific F/P-dKO males were obtained at a frequency of 1/4 by crossing *Pten*^loxP/loxP^/*Fasn*^loxP/loxP^ PbCre^neg^ females with *Pten*^loxP/loxP^/*Fasn*^loxP/loxP^ PbCre^pos^ males.

**
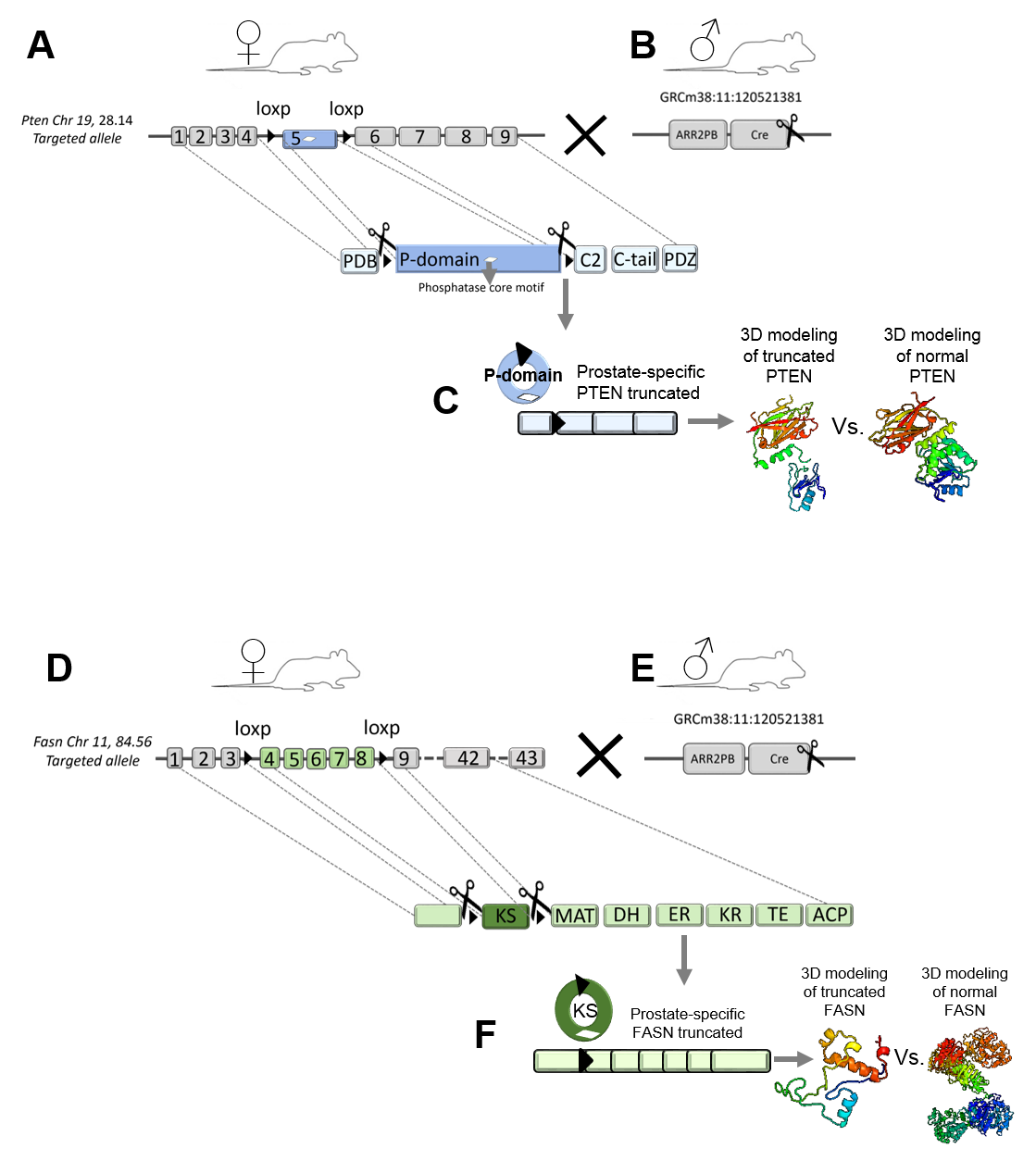
Figure S2.** *Pten* and *Fasn* prostate-specific deletion. (A) The phosphatase domain of Pten was deleted by lox-flanking exon 5 of Pten in C57Bl/6 female mice and crossing with ARR2PbCre C57Bl/6 males (B). Prostate-specific Pten inactivation occurs due to the formation of a truncated Pten (C). PDB = PIP2-binding motif; P-domain = phosphatase domain; PDZ = PDZ domain-binding motif. (D) The β-ketoacyl-synthase domain of *Fasn* was partially deleted by lox-flanking exons 4–8 (808–1556 bp) of *Fasn* in C57Bl/6 female mice and crossing with ARR2PbCre C57Bl/6 males (E). Prostate-specific deletion of exons 4–8 results in truncated *Fasn* by the insertion of a stop codon at 1592 bp (F). Truncated Fasn has 104 aa, whereas the murine Fasn has about 2500 aa.


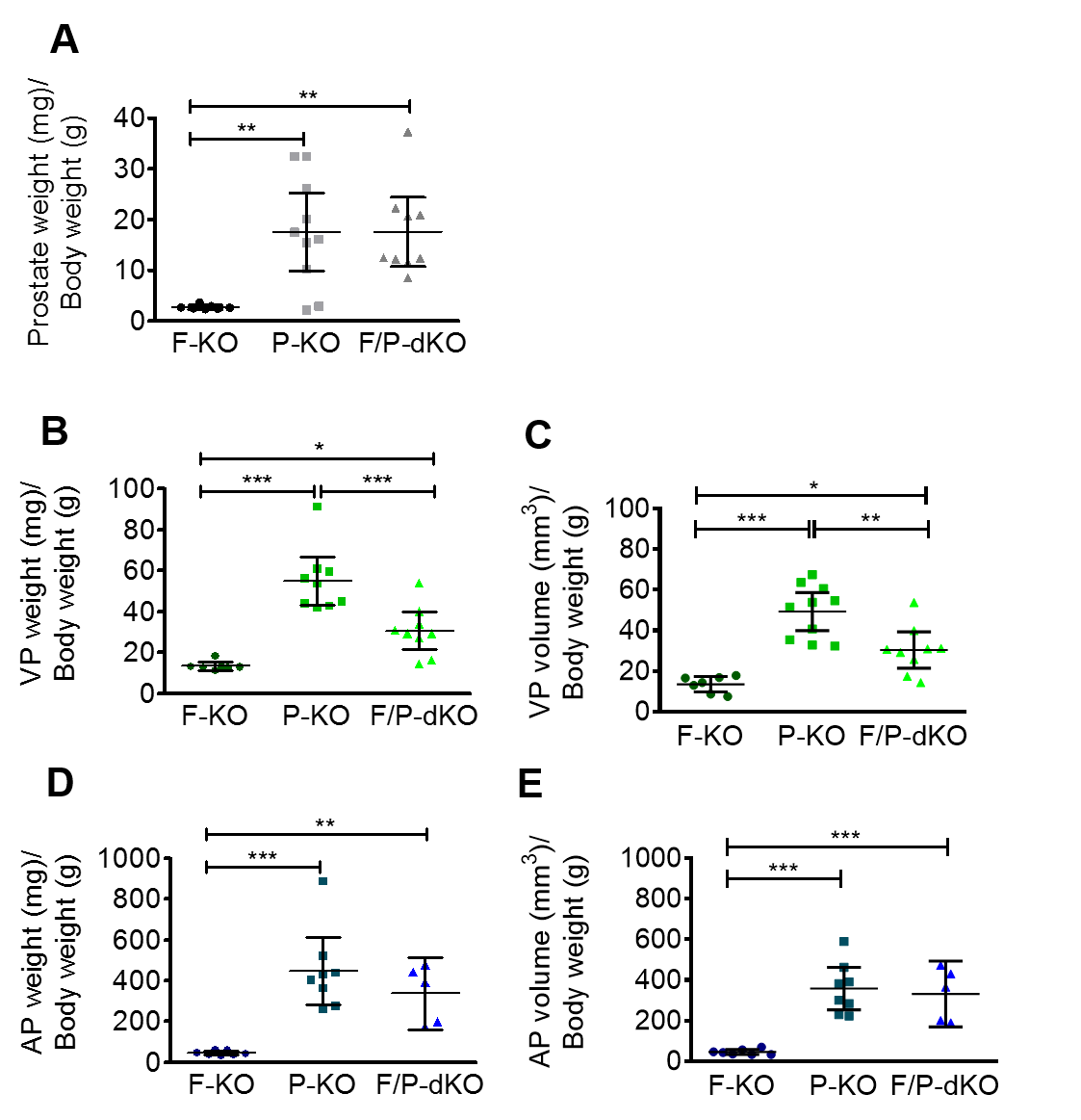


**Figure S3.** (A) Prostate weights, volumes, and stromal areas at 40 weeks. The weight of whole prostates was not affected by *Fasn* prostate-specific deletion in 40-week-old mice. Ventral prostate weight (B) and volumes (C) of 40-week-old F/P-dKO were significantly reduced in comparison with ventral prostates of P-KO mice. However, anterior prostate weight (D) and volumes (E) of 40-week-old F/P-dKO were not significantly reduced by the *Fasn* deletion in comparison with anterior prostates of P-KO mice. Prostate weights were normalized by the body weight. **p*<0.01, ***p*<0.001, ****p*<0.0001; ANOVA and Tukey’s test. Error bars indicates mean±SD of 15 mice for 7–10 mice for the 40-weeks group.

**
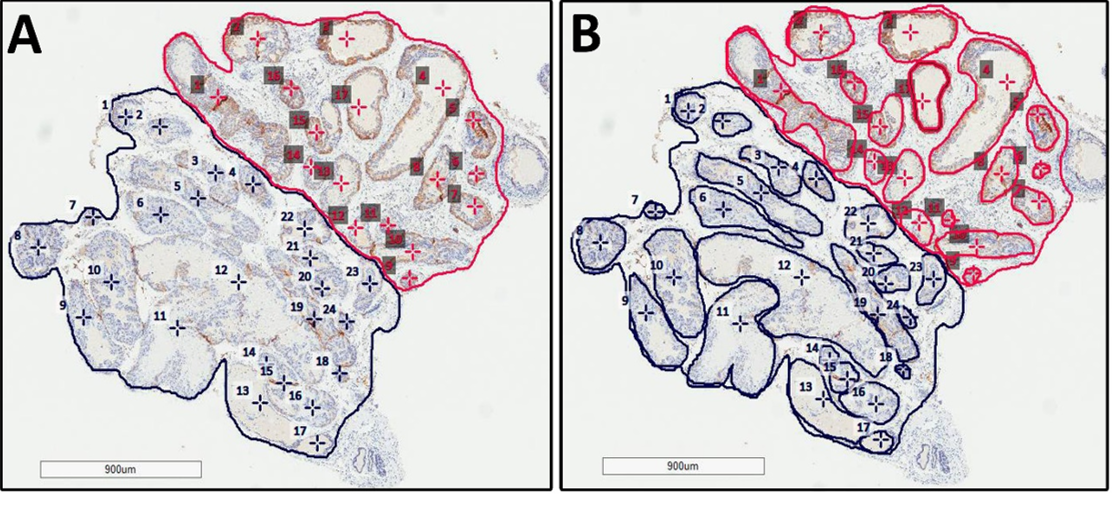
**

**Figure S4**. Discriminating Fasn-positive and -negative regions. VP of the F/P-dKO group showing areas with high positivity for Fasn (upper region) and areas of low or no positivity (lower region) for Fasn (A). (A) Acini containing 0–50% (blue) or 50–100% (pink) of cells positive for Fasn were counted, and the area of regions 0–50% positive for Fasn or 50–100% positive for Fasn was delimitated. (B) The area of each acinus was delimitated and the area calculated using Scion Image software.

**
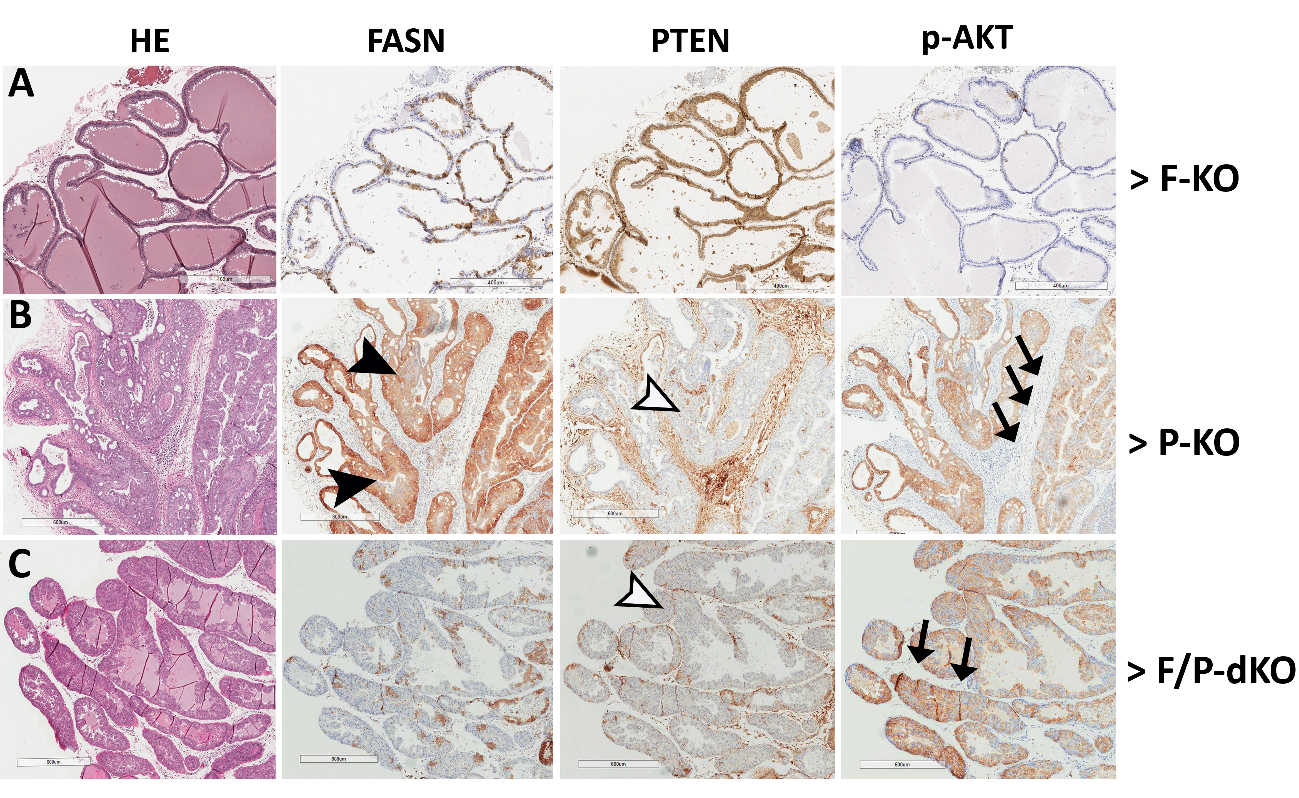
**

**Figure S5.** **Histological and immunohistochemical aspects of 40-week-old prostates.** (A) F-KO prostates showing normal aspect in H&E, chimeric loss of FASN, presence of PTEN, and no activation of p-Akt. (B) Representative H&E and IHC staining images of the ventral prostate of 40-week-old P-KO mice showing increased number of epithelial cells in the acini and enlarged stroma. (C) Representative H&E and IHC staining image of the ventral prostate of 40-week-old F/P-dKO mice showing a less aggressive phenotype than that of P-KO prostate tumors. High levels of Fasn (black arrowheads) were found in the ventral prostates of P-KO mice (B). Pten was deleted in the epithelial cells (white arrowheads), but not in the stromal cells of either P-KO or F/P-dKO mice (B, C). High levels of p-Akt were found in epithelial cells, but not in the stromal cells of P-KO and FP-dKO groups (black arrows).

**Supplementary Table S1**

**Table S1.** Summary of the primers and PCR conditions used

| **Gene** |  | **Primers (5'–3')** | **PCR conditions*** |
| --- | --- | --- | --- |
| *Pten*^loxP^ | F | TGTTTTTGACCAATTAAAGTAGGCTGTG | 95 °C – 15' [30 cycles: 95 °C – 30'',  57 °C – 1', 72^o^C – 1'] 72 °C – 5'; 4 °C ∞ |
|  | R | AAAAGTTCCCCTGCTGATGATTTGT |  |
| *Fasn*^loxP^ | F | GGATAGCTGTGTAGTGTAACCAT | 94 °C –15' [30 cycles: 94 °C – 45'',  60 °C – 45'', 72 °C – 45''] 72 °C – 10'; 4 °C ∞ |
|  | R | GGTCATCGAGACAACCACACAT |  |
| *PbCre*^loxP^ | F | TGATGGACATGTTCAGGGATC | Same for *Pten*^loxP^ or *Fasn*^loxP^ |
|  | R | CAGCCACCAGCTTGCATGA |  |

*Single prime indicates minutes and double prime seconds.
